# Angiotensin-II stimulating vs. inhibiting antihypertensive drugs and the risk of Alzheimer's disease or related dementia in a large cohort of older patients with colorectal cancer

**DOI:** 10.3389/fcvm.2023.1136475

**Published:** 2023-05-05

**Authors:** Xianglin L. Du, Zhuoyun Li, Paul E. Schulz

**Affiliations:** ^1^ Department of Epidemiology, Human Genetics and Environmental Sciences, School of Public Health, The University of Texas Health Science Center at Houston, Houston, TX, United States; ^2^ Department of Neurology, The University of Texas Health Science Center at Houston, Houston, TX, United States

**Keywords:** Alzheimer's disease, dementia, antihypertensive drugs, colorectal cancer, medicare

## Abstract

**Background:**

Several previous studies showed that patients who received angiotensin II–stimulating antihypertensive medications had a lower incident dementia rate than those angiotensin II–inhibiting antihypertensive users, but no study has been conducted in long-term cancer survivors.

**Objectives:**

To determine the risk of Alzheimer's disease (AD) and related dementia (ADRD) associated with the types of antihypertensive medications in a large cohort of survivors with colorectal cancer in 2007–2015 with follow-up from 2007 to 2016.

**Methods:**

We identified 58,699 men and women with colorectal cancer aged 65 or older from the Surveillance, Epidemiology, and End Results (SEER)—Medicare linked database in 17 SEER areas in 2007–2015 with follow-up to 2016, who were free of any diagnosed ADRD at the baseline (within 12 months prior to and 12 months after the date of diagnosis for colorectal cancer). All patients who were defined as having hypertension by ICD diagnosis code or received antihypertensive drugs during this baseline 2-year period were classified into 6 groups based on whether they received angiotensin-II stimulating or inhibiting antihypertensive drugs.

**Results:**

Crude cumulative incidence rates of AD and ADRD were similar between those who received angiotensin II–stimulating antihypertensive medications (4.3% and 21.7%) and those receiving angiotensin II–inhibiting antihypertensive medications (4.2% and 23.5%). As compared to patients who received angiotensin II–stimulating antihypertensive drugs, those who received angiotensin II–inhibiting antihypertensives were significantly more likely to develop AD (adjusted hazard ratio: 1.15, 95% CI: 1.01–1.32), vascular dementias (1.27, 1.06–1.53), and total ADRD (1.21, 1.14–1.28) after adjusting for potential confounders. These results remained similar after adjusting for medication adherence and considering death as a competing risk.

**Conclusions:**

The risk of AD and ADRD in patients with hypertension who received angiotensin II–inhibiting antihypertensive medications was higher than in those receiving angiotensin II–stimulating antihypertensive drugs in patients with colorectal cancer.

## Introduction

Although the etiologies of Alzheimer's disease (AD) and related dementia (ADRD) are still largely unknown, many vascular diseases such as cardiovascular diseases (CVD), stroke, hypertension, and diabetes have been well documented to be major risk factors for ADRD (1–5). A lower blood pressure in population-based studies (6–8) or intensive blood pressure control in a clinical trial (9) was associated with a reduced risk of cognitive impairment. Previous studies have even shown that some classes of antihypertensive drugs reduce the risk of ADRD beyond their effects on reducing blood pressure (10–27). Antihypertensive medications work through multiple different mechanisms. Two important categories, divided by mechanisms, are angiotensin-II stimulating antihypertensives (angiotensin-II receptor blockers, dihydropyridine calcium channel blocker, or thiazide-type diuretics) and angiotensin-II inhibiting antihypertensives (angiotensin-converting enzyme inhibitor, *β*-blockers, or non-dihydropyridine calcium channel blocker). The “angiotensin hypothesis” suggests that the angiotensin-II stimulating medications may improve dementia outcomes due to increasing blood flow and other potential mechanisms (10, 11, 28–31). van Dalen and colleagues (10) studied 1,909 community-dwelling individuals aged 70–78 years who participated in the Prevention of Dementia by Intensive Vascular Care (PreDIVA) trial in the Netherlands from 2006 to 2009 with 6–8 years of follow-up, and showed, in fact, that angiotensin-II stimulating antihypertensive users had a 45% lower incident dementia rate (hazard ratio: 0.55; 95% CI: 0.34–0.89) than those angiotensin-II inhibiting antihypertensive users. Marcum and colleagues (11) did a secondary analysis of participants aged ≥50 years with hypertension in the randomized Systolic Blood Pressure Intervention Trial (SPRINT) in 2011–2018 with a median of 4.8 years of follow-up, and also found that the risk of amnestic mild cognitive impairment (MCI) was significantly lower (hazard ratio: 0.74; 95% CI: 0.64–0.87) and probable dementia was insignificantly lower (0.80; 0.57–1.14) in those receiving stimulating-only vs. those inhibiting-only users. These associations between angiotensin-II stimulating antihypertensive users and a lower risk of ADRD were also reported in other studies (12–27).

Previous animal and mechanistic studies demonstrated that angiotensin-II stimulating antihypertensive medications may promote beneficial effects on the brain possibly through reduced ischemia, enhance cerebral blood flow, and improve spatial memory processing (10, 11, 28–31). No study has been conducted on this association between these classes of antihypertensive medications and the risk of ADRD in long-term cancer survivors. Patients with cancer have had many known factors that could affect the risk of dementia, such as cancer and cancer chemotherapy (5). Hence, examining this research question in patients with colorectal cancer would be interesting and important. Furthermore, with the availability of comprehensive Medicare Part-D drug data, this study aimed to determine the risk of AD and ADRD in association with the various types of antihypertensive medications in a large cohort of long-term survivors with colorectal cancer in 2007–2015 with follow-up from 2007 to 2016. We hypothesized that the risk of AD and ADRD in those with hypertension at the time of cancer diagnosis who received angiotensin-II inhibiting antihypertensive medications would be higher than in those receiving angiotensin-II stimulating antihypertensive drugs. Furthermore, this study examined the effects of antihypertensive adherence on the risk of ADRD among patients receiving various antihypertensive medications, thus making unique contributions to the literature.

## Methods

### Data sources

This study utilized the Surveillance, Epidemiology, and End Results (SEER)-Medicare linked database (32, 33) for patients with colorectal cancer at age ≥65 years in 17 SEER areas between 2007 and 2015 with follow-up from 2007 to 2016. The population covered by 17 SEER areas accounted for 28% of the U.S. population (32). The study was approved by the Committee for the Protection of Human Subjects at the University of Texas Health Science Center at Houston.

### Study design and population

**Figure 1:**
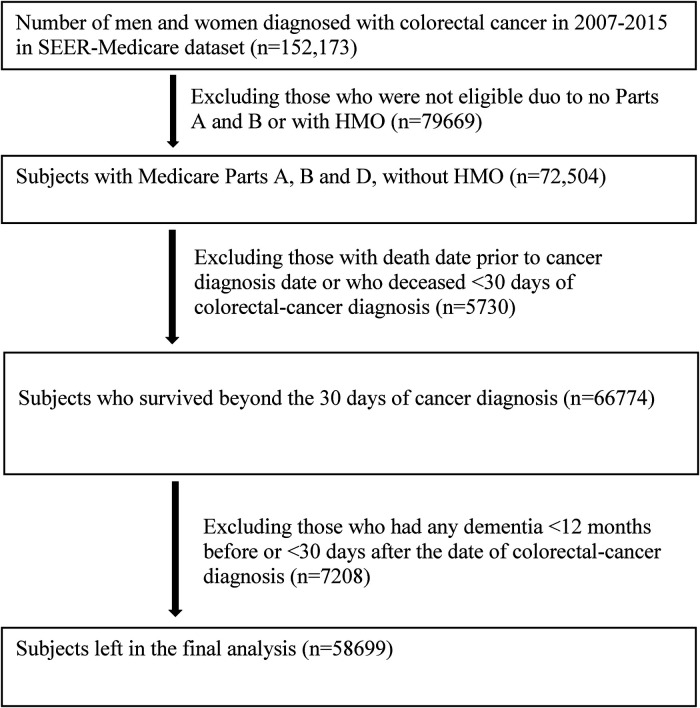
Chart of study population with inclusion and exclusion.

This is a retrospective cohort study. The study population consisted of 152,173 patients who were diagnosed with colorectal cancer at age ≥65 years between January 1, 2007 and December 31, 2015. Patients were excluded for not having Parts A and B, enrollment in a Health Maintenance Organization and Part C (Medicare Advantage), and death within 30 days of cancer diagnosis. Flowchart for inclusion and exclusion is shown in Figure 1. After exclusions, 58,699 men and women with colorectal cancer, who were free of any diagnosed ADRD at the baseline (within 12 months prior to and 12 months after the date of diagnosis for colorectal cancer), were included in the final analyses.

### Study variables

#### Main exposures

Main exposures were hypertension diagnosis and antihypertensive drug types. Hypertension was defined as having an ICD-9 diagnosis code of 401, 402, 403, 404 or 405, or having an ICD-10 diagnosis code of I10, I11, I12, I13, or I15 in Medicare data (inpatient, outpatient and physician claim files), or if antihypertensive medications were received according to Medicare Part-D drug files (Supplementary Table S1) within 12 months prior to or 12 months after the date of cancer diagnosis (i.e., within a period of 2 years). All patients were then classified into one of the following 6 groups according to antihypertensive medications received: 1. Angiotensin-II stimulating drugs (angiotensin II receptor blockers, dihydropyridine calcium channel blocker, or thiazide-type diuretics), 2. Angiotensin-II inhibiting drugs (angiotensin-converting enzyme inhibitor, *β*-blockers, or non-dihydropyridine calcium channel blocker), 3. Both Angiotensin-II stimulating and inhibiting drugs, 4. Other antihypertensive drugs, 5. Did not receive any antihypertensives, and 6. Did not have hypertension. We further defined a high adherence to antihypertensive medications as a medication possession ratio of ≥ 80% based on the number of pills supplied over the 12-months period.

#### Main outcomes

Primary outcome was the incidence of AD and the secondary outcomes were the incidence of other types of ADRD and overall ADRD from the baseline to the last date of follow-up (December 31, 2016). AD and specific types of ADRD were identified from Medicare data (inpatient, outpatient and physician files) using ICD-9 or ICD-10 diagnosis codes (Supplementary Table S2). ADRD was then divided into the following 6 specific types of dementia: AD, vascular dementia, dementia with Lewy bodies (DLB), Frontotemporal degeneration and dementia (FTD), Mild cognitive impairment (MCI), and other dementia.

#### Other covariates

Other variables include sociodemographic factors (age at cancer diagnosis, gender, race and ethnicity, and marital status), tumor factors (tumor stage, grade, site, and receipt of chemotherapy and radiotherapy), comorbidity score, calendar year of cancer diagnosis (2007–2015), and SEER areas by state where the registries are located (32, 33). Race/ethnicity was coded as Non-Hispanic [NH]-whites, NH-blacks, NH-Asians/Pacific Islanders, Hispanics, others, or unknown/missing. Comorbidities were defined as co-existing medical conditions other than the interest under study (ADRD, hypertension, and cancer). These included myocardial infarction, congestive heart failure, peripheral vascular disease, cerebrovascular disease, chronic pulmonary disease, congestive tissue disease, ulcer disease, mild liver disease, diabetes, hemiplegia, moderate or severe renal disease, diabetes with end organ damage, leukemia, lymphoma, moderate or severe liver disease, and human immunodeficiency virus (HIV) positive or acquired immune deficiency syndrome (AIDS) (34–37). Comorbidities were identified through diagnoses or procedures made 1 year prior to and 1 year after the date of cancer diagnosis using SAS programs provided by the National Cancer Institute (37). Each comorbid disease was weighted according to the severity of comorbid conditions (34–37) and the sum of all scores were categorized as 0, 1, and ≥2.

### Analysis

The distributions of baseline characteristics between colorectal cancer patients by antihypertensive medications were compared using the chi-square statistic for categorical variables or using the Kruskal-Wallis test for median age comparisons. Cumulative incidence of ADRD was defined as the ratio of the number of cases with a new ADRD over the total number of participants-at risk who were free of any diagnosed ADRD at the baseline when a colorectal cancer diagnosis was made. Incidence density was defined as the ratio of the number of cases with a new ADRD diagnosis over the total number of person-years by taking into consideration the differential follow-up times of study participants. This study used the Cox regression models for the time to event analysis to determine the risk of developing ADRD by exposures while adjusting for potential confounders. The proportionality assumption was evaluated by the log-log Kaplan-Meier curves and interaction terms between exposures and time variables in the Cox regression models (38). The Fine and Gray competing risk proportional hazards regression was analyzed by considering death as a competing risk (39). A *p* value of <0.05 was considered statistically significant. Analyses were conducted using SAS version 9.4 (Cary, NC: SAS Institute, Inc.) and R version 4.0.2 (R Foundation for Statistical Computing).

## Results

### Baseline characteristics by main exposure

Table 1 presents the distribution of baseline characteristics by hypertension status and antihypertensive medication types. The median age was the lowest at 73 years among those without hypertension and the highest at 79 years in those who received other hypertensive medications. The median age for patients receiving angiotensin-II stimulating and inhibiting antihypertensive medications was 77 and 76, respectively. Patients receiving angiotensin-II stimulating and inhibiting antihypertensive medications exhibited small differences in distribution by age groups, whereas those without hypertension had a higher proportion of younger patients aged 65–69 years (30.5%). The distribution of other socio-demographic factors, tumor characteristics and year of diagnosis also varied by hypertension and medication types.

**Table 1:** Distributions of baseline characteristics in men and women with colorectal cancer by antihypertensive drug types.

	Number of cases (column %) by antihypertensive drug types						
Patient and Tumor Characteristics	Angiotensin-II stimulating drugs	Angiotensin-II inhibiting drugs	Angiotensin-II stimulating + inhibiting drugs	Other anti-hypertensive drugs	Did not receive any drugs for hypertension	No hypertension diagnosed	*P* Value^a^
Median age (range)	77 (65–102)	76 (65–108)	76 (65–101)	79 (65–108)	77 (65–104)	73 (65–105)	<0.001
Age (years)							
65–69	1,560 (19.7)	3,077 (21.9)	2,489 (21.6)	712 (15.7)	2,494 (20.5)	2,593 (30.5)	<0.001
70–74	1,678 (21.1)	3,071 (21.9)	2,543 (22.1)	785 (17.3)	2,450 (20.2)	2,004 (23.6)	
75–79	1,584 (20.0)	2,761 (19.7)	2,429 (21.1)	908 (20.0)	2,332 (19.2)	1,553 (18.3)	
80–84	1,554 (19.6)	2,541 (18.1)	2,095 (18.2)	932 (20.5)	2,201 (18.1)	1,158 (13.6)	
85 or older	1,561 (19.7)	2,602 (18.5)	1,948 (16.9)	1,208 (26.6)	2,681 (22.1)	1,195 (14.1)	
Gender							
Men	2,956 (37.2)	6,836 (48.7)	4,712 (41.0)	1,863 (41.0)	5,916 (48.7)	4,211 (49.5)	<0.001
Women	4,981 (62.8)	7,216 (51.4)	6,792 (59.0)	2,682 (59.0)	6,242 (51.3)	4,292 (50.5)	
Race/ethnicity							
NH-Whites	5,486 (69.1)	10,740 (76.4)	8,084 (70.3)	3,648 (80.3)	9,179 (75.5)	6,471 (76.1)	<0.001
NH-Blacks	952 (12.0)	1,246 (8.9)	1,501 (13.1)	364 (8.0)	1,147 (9.4)	561 (6.6)	
NH-Asians/PIs	808 (10.2)	766 (5.5)	864 (7.5)	216 (4.8)	857 (7.1)	618 (7.3)	
Hispanics	625 (7.9)	1,175 (8.4)	976 (8.5)	286 (6.3)	867 (7.1)	768 (9.0)	
Others/unknown	66 (0.9)	125 (0.9)	79 (0.7)	31 (0.7)	108 (0.8)	85 (1.0)	
Marital status							
Married	3,367 (42.4)	6,241 (44.4)	5,026 (43.7)	1,837 (40.4)	5,618 (46.2)	4,205 (49.5)	<0.001
Unmarried	4,075 (51.3)	6,996 (49.8)	5,803 (50.4)	2,450 (53.9)	5,835 (48.0)	3,821 (44.9)	
Unknown	495 (6.2)	815 (5.8)	675 (5.9)	258 (5.7)	705 (5.8)	477 (5.6)	
Health Insurance							
Insured for private	6,246 (78.7)	11,518 (82)	8,967 (78.0)	3,704 (81.5)	10,269 (84.5)	7,372 (86.7)	<0.001
Medicaid	1,309 (16.5)	2,001 (14.2)	2,119 (18.4)	669 (14.7)	1,320 (10.9)	677 (8.0)	
Not insured/missing	382 (4.8)	533 (3.8)	418 (3.6)	172 (3.8)	569 (4.7)	454 (5.3)	
Tumor stage							
In-situ/local stage	3,629 (45.7)	6,270 (44.6)	5,781 (50.3)	1,698 (37.4)	4,426 (36.4)	3,037 (35.7)	<0.001
Regional stage	2,509 (31.6)	4,712 (33.5)	3,825 (33.3)	1,467 (32.3)	3,793 (31.2)	2,520 (29.6)	
Distant stage	1,432 (18.0)	2,445 (17.4)	1,453 (12.6)	1,091 (24.0)	3,179 (26.2)	2,413 (28.4)	
Unknown/missing	367 (4.6)	625 (4.5)	445 (3.9)	289 (6.4)	760 (6.3)	533 (6.3)	
Tumor size (cm)							
<1	333 (4.2)	554 (3.9)	498 (4.3)	153 (3.4)	427 (3.5)	319 (3.8)	<0.001
1-<2	450 (5.7)	798 (5.7)	693 (6.0)	195 (4.3)	630 (5.2)	386 (4.5)	
2-<3	797 (10.0)	1,343 (9.6)	1,313 (11.4)	345 (7.6)	991 (8.2)	646 (7.6)	
3-<4	1,074 (13.5)	1,881 (13.4)	1,693 (14.7)	576 (12.7)	1,397 (11.5)	926 (10.9)	
≥4	3,456 (43.5)	6,482 (46.1)	4,821 (41.9)	2,159 (47.5)	5,608 (46.1)	3,875 (45.6)	
Missing	1,827 (23.0)	2,994 (21.3)	2,486 (21.6)	1,117 (24.6)	3,105 (25.5)	2,351 (27.7)	
Tumor grade							
Well-differentiated	609 (7.7)	1,135 (8.1)	1,000 (8.7)	342 (7.5)	910 (7.5)	603 (7.1)	<0.001
Moderately-differentiated	4,630 (58.3)	8,213 (58.5)	6,877 (59.8)	2,486 (54.7)	6,595 (54.2)	4,515 (53.1)	
Poorly-differentiated	1,365 (17.2)	2,438 (17.4)	1,850 (16.1)	855 (18.8)	2,348 (19.3)	1,537 (18.1)	
Unknown/missing	1,333 (16.8)	2,266 (16.1)	1,777 (15.5)	862 (19.0)	2,305 (19.0)	1,848 (21.7)	
Tumor site							
Colon	6,047 (76.2)	10,645 (75.8)	8,839 (76.8)	3,604 (79.3)	9,114 (75.0)	6,103 (71.8)	<0.001
Rectal	1,890 (23.8)	3,407 (24.3)	2,665 (23.2)	941 (20.7)	3,044 (25.0)	2,400 (28.2)	
Comorbidity scores							
0	3,684 (46.4)	6,471 (46.1)	5,042 (43.8)	1,690 (37.2)	5,685 (46.8)	5,780 (68.0)	<0.001
1	2,761 (34.8)	4,759 (33.9)	3,890 (33.8)	1,779 (39.1)	4,105 (33.8)	2,115 (24.9)	
≥2	1,492 (18.8)	2,822 (20.1)	2,572 (22.4)	1,076 (23.7)	2,368 (19.5)	608 (7.2)	
Year of Diagnosis							
2007	914 (11.5)	1,321 (9.4)	1,975 (17.2)	374 (8.2)	1,198 (9.9)	986 (11.6)	<0.001
2008	957 (12.1)	1408 (10.0)	1,714 (14.9)	455 (10.0)	1,181 (9.7)	935 (11.0)	
2009	894 (11.3)	1,433 (10.2)	1,599 (13.9)	451 (9.9)	1,172 (9.6)	874 (10.3)	
2010	892 (11.2)	1,453 (10.3)	1,374 (11.9)	468 (10.3)	1,202 (9.9)	832 (9.8)	
2011	933 (11.8)	1,580 (11.2)	1,247 (10.8)	489 (10.8)	1,274 (10.5)	868 (10.2)	
2012	903 (11.4)	1,764 (12.6)	1,107 (9.6)	599 (13.2)	1,504 (12.4)	989 (11.6)	
2013	859 (10.8)	1,712 (12.2)	985 (8.6)	589 (13.0)	1,469 (12.1)	1,010 (11.9)	
2014	851 (10.7)	1,716 (12.2)	835 (7.3)	584 (12.9)	1,548 (12.7)	981 (11.5)	
2015	734 (9.3)	1,665 (11.9)	668 (5.8)	536 (11.8)	1,610 (13.2)	1,028 (12.1)	
Total	7,937 (100.0)	14,052 (100.0)	11,504 (100.0)	4,545 (100.0)	12,158 (100.0)	8,503 (100.0)	

^a^ *P* values were from Kruskal-Wallis Test for median ages and from the chi-square test for categorical variables.

### Cumulative incidence of ADRD

Table 2 presents the cumulative incidence of ADRD in colorectal cancer patients with up to 10 years of follow-up from 2007 to 2016 by antihypertensive medication types. The crude cumulative incidence rates of AD and ADRD were comparable in those who received angiotensin-II stimulating antihypertensive medications (4.3% and 21.7%) and those receiving angiotensin-II inhibiting medications (4.2% and 23.5%). The cumulative incidence rates of AD and ADRD were the highest in those receiving the combination of angiotensin-II stimulating and inhibiting antihypertensive drugs (6.1% and 28.2%) and the lowest in those without hypertension (1.9% and 11.2%). Cumulative incidence rates of AD and other types of ADRD increased substantially with age, were slightly higher in women, and varied by race/ethnicity with a higher incidence in NH-blacks. Cumulative incidence rates of ADRD also slightly varied by tumor factors and cancer treatments and increased with comorbidity scores. Similar patterns were observed in the incidence density rates of AD and other ADRD types (Supplementary Table S3).

**Table 2:** Cumulative-incidence of dementia (ADRD^a^) by antihypertensive drug types.

Characteristics	Cumulative-incidence (%) of ADRD^a^						
	AD^a^	Vascular	DLB^a^	FTD^a^	MCI^a^	Others	Total
Antihypertensive drug types							
Angiotensin-II stimulating drugs	4.3	2.1	0.3	0.2	1.5	20.3	21.7
Angiotensin-II inhibiting drugs	4.2	2.4	0.4	0.1	1.7	22.1	23.5
Angiotensin-II stimulating + inhibiting drugs	6.1	3.2	0.4	0.2	2.1	26.5	28.2
Other antihypertensives	4.0	2.6	0.3	0.1	1.6	23.7	25.1
Did not receive antihypertensives	2.4	1.4	0.3	0	1.1	17.5	18.6
Did not have hypertension	1.9	0.6	0.2	0.1	0.9	10.4	11.2
Age (years)							
65–69	1.6	1.1	0.2	0	0.9	10.5	11.4
70–74	2.6	1.5	0.3	0.1	1.2	15.2	16.4
75–79	4.3	2.3	0.4	0.1	1.6	20.7	22.2
80–84	5.7	2.9	0.4	0.1	1.9	26.4	27.7
85 or older	5.7	2.9	0.3	0.1	2.1	30.8	32.4
Gender							
Men	3.1	1.8	0.4	0.1	1.3	18.1	19.4
Women	4.5	2.3	0.3	0.1	1.6	22.0	23.3
Race/ethnicity							
NH-Whites	3.9	2.0	0.3	0.1	1.6	20.3	21.6
NH-Blacks	4.3	3.2	0.2	0.1	1.4	22.8	24.3
NH-Asians/Pacific Islanders	3.5	1.6	0.3	0	0.9	18.9	19.9
Hispanics	4.0	1.9	0.3	0.1	0.9	18.5	19.8
Others	0.8	1.2	0	0.4	1.2	14.6	15.0
Unknown/missing	2.1	1.3	0	0.4	1.3	12.1	12.9
Marital status							
Married	3.3	1.6	0.3	0.1	1.4	16.6	17.8
Unmarried	4.3	2.5	0.3	0.1	1.6	23.5	24.9
Unknown	4.1	1.9	0.3	0.1	1.6	20.1	21.4
Tumor stage							
In-situ/local stage	5.4	2.7	0.5	0.1	1.9	23.0	24.6
Regional	4.0	2.2	0.3	0.1	1.6	21.3	22.8
Distant	0.6	0.7	0	0	0.5	12.0	12.5
Unknown/Missing	3.0	2.0	0.2	0.2	1.1	22.9	24.0
Tumor grade							
Well-differentiated	4.7	2.1	0.3	0.2	1.8	21.0	22.7
Moderately-differentiated	4.3	2.2	0.4	0.1	1.5	21.1	22.4
Poorly-differentiated	3.2	1.9	0.2	0.1	1.6	19.3	20.6
Unknown/Missing	3.0	1.9	0.3	0.1	1.1	18.0	19.3
Tumor site							
Colon	4.1	2.2	0.3	0.1	1.6	21.0	22.3
Rectal	3.2	1.6	0.3	0.2	1.2	17.9	19.0
Chemotherapy							
No	4.4	2.4	0.4	0.1	1.6	21.8	23.2
Yes	2.3	1.1	0.2	0.1	1.2	15.4	16.5
Radiotherapy							
No	4.0	2.1	0.3	0.1	1.5	20.5	21.8
Yes	2.9	1.3	0.2	0.1	1.0	16.7	18.1
Comorbidity Scores							
0	3.4	1.7	0.3	0.1	1.4	16.3	17.5
1	4.4	2.4	0.4	0.1	1.6	22.2	23.6
≥2	4.1	2.6	0.2	0.1	1.5	26.8	28.3
Total	3.9	2.1	0.3	0.1	1.5	20.2	21.5

^a^ ADRD, Alzheimer's disease and related dementia; AD, Alzheimer's disease; Vascular, vascular dementia; DLB, dementia with Lewy bodies; FTD, Frontotemporal degeneration and dementia; MCI, Mild cognitive impairment; others (other dementia), and total ADRD.

### Hazard ratio of developing ADRD by main exposures and other factors

Table 3 presents the hazard ratio of AD and other types of ADRD by antihypertensive medication types after adjusting for patient age, gender, race/ethnicity, marital status, comorbidity, tumor factors, year of diagnosis, and geographic (SEER) areas. As compared to patients who received angiotensin-II stimulating antihypertensive drugs, those who received angiotensin-II inhibiting drugs were significantly more likely to develop AD (adjusted hazard ratio: 1.15, 95% CI: 1.01–1.32), vascular dementia (1.27, 1.06–1.53), MCI (1.27, 1.02–1.59), other dementia (1.22, 1.15–1.30), and total ADRD (1.21, 1.14–1.28), but had no significantly different risk of DLB and FTD. Those receiving a combination of angiotensin-II stimulating and inhibiting antihypertensive drugs did not have significantly different risks of AD, vascular dementia, DLB, FTD, and MCI, but had a significantly higher risk of other dementia and total ADRD. Patients who received other types of antihypertensive drugs had a significantly higher risk of AD, vascular dementia, other dementia, and total ADRD, but had no significantly different risk for DLB, FTD, and MCI. Patients with hypertension who did not take antihypertensive medications had a significantly higher risk of other dementia, and total ADRD only, whereas those without hypertension had a significantly lower risk of vascular dementia (0.53, 0.39–0.71), other dementia (0.80, 0.73–0.87), and total ADRD (0.79, 0.73–0.86). A forest plot is presented in Supplementary Figure S1 for the hazard ratio of developing AD and other types of dementia between patients receiving angiotensin II–stimulating antihypertensive medications and those receiving angiotensin II–inhibiting antihypertensive medications, indicating a statistically significant difference in the risk of AD, vascular dementia, MCI, other dementia, and total ADRD.

**Table 3:** Adjusted hazard ratio of developing dementia by antihypertensive drug types.

	Hazard ratio (95% confidence intervals)^a^ of ADRD^b^						
Characteristics	AD^a^	Vascular	DLB^a^	FTD^a^	MCI^a^	Others	Total
Antihypertensive drug types							
Angiotensin-II stimulating drugs	1.0 (ref)	1.0 (ref)	1.0 (ref)	1.0 (ref)	1.0 (ref)	1.0 (ref)	1.0 (ref)
Angiotensin-II inhibiting drugs	1.15 (1.01–1.32)	1.27 (1.06–1.53)	1.35 (0.83–2.17)	0.77 (0.36–1.64)	1.27 (1.02–1.59)	1.22 (1.15–1.30)	1.21 (1.14–1.28)
Angiotensin -II stimulating + inhibiting drugs	1.04 (0.92–1.19)	1.17 (0.97–1.41)	0.87 (0.53–1.43)	0.83 (0.40–1.72)	1.15 (0.92–1.44)	1.09 (1.02–1.16)	1.09 (1.02–1.15)
Other antihypertensives	1.26 (1.05–1.52)	1.52 (1.20–1.93)	1.37 (0.71–2.65)	0.49 (0.14–1.74)	1.29 (0.96–1.73)	1.32 (1.23–1.43)	1.32 (1.22–1.42)
Did not receive antihypertensives	1.15 (0.98–1.35)	1.13 (0.91–1.40)	1.46 (0.86–2.48)	0.50 (0.19–1.35)	1.18 (0.92–1.52)	1.29 (1.21–1.38)	1.28 (1.20–1.36)
Did not have hypertension	0.83 (0.69–1.01)	0.53 (0.39–0.71)	1.10 (0.60–2.00)	0.71 (0.26–1.93)	0.88 (0.65–1.18)	0.80 (0.73–0.87)	0.79 (0.73–0.86)
Age (years)							
65–69	1.0 (ref)	1.0 (ref)	1.0 (ref)	1.0 (ref)	1.0 (ref)	1.0 (ref)	1.0 (ref)
70–74	1.75 (1.47–2.08)	1.39 (1.11–1.73)	1.98 (1.15–3.42)	4.04 (1.34–12.21)	1.35 (1.06–1.72)	1.53 (1.43–1.65)	1.52 (1.42–1.63)
75–79	3.11 (2.64–3.67)	2.26 (1.84–2.78)	3.52 (2.09–5.92)	5.27 (1.75–15.89)	1.98 (1.57–2.51)	2.27 (2.12–2.43)	2.24 (2.10–2.39)
80–84	4.77 (4.05–5.61)	2.93 (2.38–3.61)	3.67 (2.13–6.34)	5.51 (1.76–17.22)	2.50 (1.97–3.17)	3.25 (3.04–3.47)	3.14 (2.94–3.35)
85 or older	6.84 (5.80–8.07)	3.50 (2.83–4.32)	4.49 (2.54–7.94)	7.96 (2.50–25.29)	3.44 (2.71–4.37)	4.63 (4.33–4.95)	4.52 (4.23–4.82)
Gender							
Women vs Men	1.00 (0.91–1.10)	0.89 (0.79–1.01)	0.50 (0.36–0.68)	0.73 (0.42–1.26)	0.95 (0.82–1.10)	0.91 (0.88–0.95)	0.91 (0.88–0.95)
Race/ethnicity							
NH-Whites	1.0 (ref)	1.0 (ref)	1.0 (ref)	1.0 (ref)	1.0 (ref)	1.0 (ref)	1.0 (ref)
NH-Blacks	1.30 (1.13–1.50)	1.76 (1.48–2.09)	0.86 (0.47–1.59)	0.66 (0.23–1.91)	1.08 (0.85–1.38)	1.24 (1.17–1.32)	1.25 (1.18–1.33)
NH-Asians/Pacific Islanders	1.00 (0.83–1.21)	0.83 (0.63–1.10)	0.70 (0.37–1.33)	0.30 (0.07–1.27)	0.46 (0.32–0.66)	0.93 (0.86–1.01)	0.92 (0.85–0.99)
Hispanics	1.30 (1.11–1.53)	1.10 (0.87–1.38)	0.91 (0.52–1.59)	0.63 (0.22–1.82)	0.53 (0.39–0.72)	1.03 (0.96–1.11)	1.03 (0.96–1.10)
Others	0.38 (0.09–1.50)	0.97 (0.31–3.03)	-^c^	3.44 (0.45–26.09)	0.88 (0.28–2.77)	0.99 (0.71–1.37)	0.94 (0.68–1.30)
Unknown/missing	0.52 (0.22–1.26)	0.66 (0.21–2.08)	-	1.65 (0.20–13.51)	0.60 (0.19–1.90)	0.59 (0.41–0.85)	0.58 (0.41–0.82)
Marital status							
Married	1.0 (ref)	1.0 (ref)	1.0 (ref)	1.0 (ref)	1.0 (ref)	1.0 (ref)	1.0 (ref)
Unmarried	1.11 (1.01–1.23)	1.33 (1.17–1.52)	1.05 (0.76–1.45)	0.90 (0.51–1.59)	1.05 (0.91–1.22)	1.30 (1.24–1.35)	1.29 (1.24–1.34)
Unknown	1.00 (0.83–1.20)	0.96 (0.74–1.25)	0.94 (0.50–1.78)	0.91 (0.33–2.52)	1.08 (0.81–1.44)	1.12 (1.03–1.22)	1.11 (1.03–1.20)
Tumor stage							
Local	1.0 (ref)	1.0 (ref)	1.0 (ref)	1.0 (ref)	1.0 (ref)	1.0 (ref)	1.0 (ref)
Regional	0.99 (0.89–1.08)	1.11 (0.97–1.26)	1.02 (0.73–1.44)	1.17 (0.64–2.12)	0.99 (0.85–1.16)	1.11 (1.06–1.16)	1.11 (1.06–1.15)
Distant	0.82 (0.64–1.05)	0.97 (0.76–1.24)	0.38 (0.12–1.25)	0.29 (0.04–2.24)	0.81 (0.61–1.08)	1.38 (1.29–1.47)	1.33 (1.25–1.42)
Unknown/Missing	1.56 (1.25–1.94)	1.41 (1.07–1.86)	1.03 (0.41–2.56)	4.54 (1.88–10.95)	1.20 (0.84–1.72)	1.65 (1.52–1.79)	1.62 (1.49–1.76)
Tumor grade							
Well-differentiated	1.0 (ref)	1.0 (ref)	1.0 (ref)	1.0 (ref)	1.0 (ref)	1.0 (ref)	1.0 (ref)
Moderately-differentiated	1.04 (0.90–1.20)	1.18 (0.95–1.46)	1.37 (0.80–2.35)	0.73 (0.34–1.59)	0.98 (0.77–1.24)	1.06 (0.99–1.13)	1.05 (0.98–1.12)
Poorly-differentiated	1.01 (0.84–1.20)	1.24 (0.97–1.59)	0.90 (0.45–1.81)	0.66 (0.24–1.80)	1.24 (0.94–1.62)	1.10 (1.01–1.18)	1.10 (1.02–1.19)
Unknown/Missing	1.10 (0.93–1.32)	1.38 (1.08–1.77)	1.62 (0.87–3.04)	0.69 (0.26–1.85)	0.98 (0.73–1.30)	1.11 (1.03–1.20)	1.12 (1.03–1.21)
Tumor site							
Rectal vs Colon	0.93 (0.83–1.05)	0.85 (0.72–0.99)	1.10 (0.76–1.61)	2.05 (1.16–3.64)	0.89 (0.74–1.08)	0.98 (0.94–1.03)	0.97 (0.92–1.02)
Chemotherapy							
Yes vs No	0.74 (0.64–0.84)	0.58 (0.49–0.70)	0.70 (0.44–1.11)	1.07 (0.52–2.17)	0.92 (0.76–1.12)	0.80 (0.76–0.84)	0.80 (0.76–0.84)
Radiotherapy							
Yes vs No	1.11 (0.91–1.35)	0.97 (0.73–1.30)	0.74 (0.36–1.52)	0.60 (0.21–1.69)	0.88 (0.64–1.22)	0.98 (0.91–1.07)	1.00 (0.93–1.09)
Comorbidity Scores							
0	1.0 (ref)	1.0 (ref)	1.0 (ref)	1.0 (ref)	1.0 (ref)	1.0 (ref)	1.0 (ref)
1	1.21 (1.10–1.32)	1.31 (1.15–1.49)	1.05 (0.76–1.43)	1.42 (0.80–2.54)	1.05 (0.90–1.22)	1.33 (1.27–1.38)	1.31 (1.26–1.36)
≥2	1.50 (1.33–1.68)	1.65 (1.42–1.92)	0.89 (0.57–1.41)	2.08 (1.07–4.04)	1.27 (1.06–1.53)	1.96 (1.87–2.05)	1.93 (1.84–2.02)

^a^ Hazard ratios adjusted for the following variables: age, gender, race/ethnicity, marital status, tumor stage, tumor grade, tumor site, comorbidity score, chemotherapy, radiation therapy, year of diagnosis, and SEER areas.

^b^ ADRD, Alzheimer's disease and related dementia;, AD, Alzheimer's disease; Vascular, vascular dementia; DLB, dementia with Lewy bodies; FTD, Frontotemporal degeneration and dementia; MCI, Mild cognitive impairmen; others (other dementia), and total (any of above ADRD).

^c^ no cases.

Table 3 also presents the risk of ADRD associated with patient demographics and tumor factors. Age was significantly associated with an increased risk of all types of ADRD. For example, as compared to patients aged 65–69, those aged 75–79 years were >3 times more likely to develop AD (3.11, 2.64–3.67) and those aged ≥80 were >6 times more likely to develop AD (6.84, 5.80–8.07). Women had no significantly different risk of AD, vascular dementia, FTD, and MCI from men, but had a lower risk of DLB, other dementia and total ADRD than that of men. As compared to NH-whites, NH-black patients had a significantly higher risk of AD, vascular dementia, other dementia, and total ADRD, while Asians had a significantly lower risk of MCI and total ADRD and Hispanics had a significantly higher risk of AD. Unmarried patients had a significantly higher risk of AD, vascular dementia, other dementia, and total ADRD. There were no consistent associations between the risk of ADRD and tumor factors such as tumor stage, size, grade, and site. Chemotherapy was associated with a significantly lower risk of AD, vascular dementia, other dementia, and total ADRD, while radiation therapy was not significantly associated with the risk of any type of ADRD. Comorbidity scores of 1 and 2 or higher were associated with a significantly higher risk of AD, vascular dementia, MCI, other dementia, and total ADRD.

### Effects of adherence to medications on the risk of dementia

Table 4 further presents the effects of adherence to antihypertensive medications on the risk of dementia. As compared to patients who had a high adherence to angiotensin-II stimulating drugs, those with a low adherence had an insignificantly elevated risk of AD and total ADRD, whereas those receiving angiotensin-II inhibiting drugs had a significantly higher risk of other dementia and total ADRD regardless of their high or low adherence. Moreover, those with low adherence also had a significantly higher risk of AD (1.34, 1.09–1.65). Patients who received a combination of angiotensin-II stimulating and inhibiting drugs with high or low adherence did not have significantly different risk of AD and ADRD. In contrast, those who had a high or low adherence of other antihypertensive drugs had significantly higher risks of other dementia and total ADRD as compared to patients who had a high adherence of angiotensin-II stimulating drugs. Using the same population as a reference, those with hypertension who did not receive antihypertensive drugs were significantly more likely to develop other dementia and total ADRD but had no significantly different risk of AD and vascular dementia, whereas those without a diagnosed hypertension were significantly less likely to develop AD, vascular dementia, other dementia and total ADRD. Medication adherence in association with patient demographic and tumor factors did not alter the risk of ADRD vs. findings that only considered the broader categories of antihypertensive medications as shown in Table 3.

**Table 4:** Adjusted hazard ratio of developing dementia by adherence to antihypertensive medication use.

	Hazard ratio (95% confidence intervals)^a^ of ADRD^b^						
Characteristics	AD^a^	Vascular	DLB^a^	FTD^a^	MCI^a^	Others	Total
Antihypertensive drug types							
Angiotensin-II stimulating drugs							
With high adherence	1.0 (ref)	1.0 (ref)	1.0 (ref)	1.0 (ref)	1.0 (ref)	1.0 (ref)	1.0 (ref)
With low adherence	0.87 (0.66–1.13)	1.11 (0.77–1.59)	0.91 (0.34–2.42)	1.25 (0.34–4.62)	0.65 (0.39–1.08)	1.06 (0.94–1.19)	1.05 (0.94–1.18)
Angiotensin-II inhibiting drugs							
With high adherence	1.06 (0.91–1.24)	1.31 (1.06–1.62)	1.28 (0.75–2.19)	0.81 (0.34–1.91)	1.19 (0.93–1.52)	1.23 (1.15–1.31)	1.21 (1.13–1.29)
With low adherence	1.34 (1.09–1.65)	1.26 (0.93–1.70)	1.48 (0.72–3.04)	0.81 (0.22–3.00)	1.12 (0.79–1.60)	1.27 (1.16–1.40)	1.28 (1.17–1.41)
Angiotensin -II stimulating + inhibiting drugs							
With high adherence	1.02 (0.88–1.18)	1.16 (0.94–1.43)	0.89 (0.51–1.53)	0.80 (0.35–1.85)	1.07 (0.84–1.36)	1.10 (1.02–1.17)	1.10 (1.03–1.17)
With low adherence	0.98 (0.78–1.23)	1.41 (1.04–1.90)	0.66 (0.25–1.77)	1.32 (0.40–4.33)	1.06 (0.71–1.57)	1.13 (1.01–1.26)	1.11 (1.00–1.23)
Other antihypertensives							
With high adherence	1.34 (1.08–1.68)	1.75 (1.32–2.33)	1.19 (0.50–2.82)	0.29 (0.04–2.31)	1.30 (0.91–1.84)	1.48 (1.35–1.63)	1.46 (1.34–1.61)
With low adherence	1.07 (0.82–1.40)	1.30 (0.92–1.84)	1.55 (0.65–3.68)	0.82 (0.18–3.83)	1.06 (0.70–1.62)	1.16 (1.04–1.30)	1.16 (1.04–1.29)
Did not receive antihypertensives	1.12 (0.95–1.32)	1.15 (0.91–1.45)	1.43 (0.81–2.52)	0.53 (0.19–1.50)	1.09 (0.84–1.42)	1.30 (1.22–1.40)	1.29 (1.21–1.38)
Did not have hypertension	0.81 (0.66–0.98)	0.54 (0.39–0.74)	1.08 (0.57–2.02)	0.75 (0.26–2.14)	0.81 (0.60–1.10)	0.81 (0.74–0.88)	0.80 (0.73–0.87)
Age (years)							
65–69	1.0 (ref)	1.0 (ref)	1.0 (ref)	1.0 (ref)	1.0 (ref)	1.0 (ref)	1.0 (ref)
70–74	1.75 (1.47–2.08)	1.39 (1.12–1.74)	1.97 (1.14–3.42)	4.04 (1.34–12.21)	1.35 (1.06–1.72)	1.54 (1.43–1.65)	1.52 (1.42–1.63)
75–79	3.12 (2.65–3.67)	2.26 (1.83–2.78)	3.52 (2.09–5.92)	5.27 (1.75–15.90)	1.98 (1.56–2.50)	2.27 (2.13–2.43)	2.24 (2.10–2.39)
80–84	4.77 (4.06–5.61)	2.94 (2.39–3.61)	3.67 (2.13–6.33)	5.53 (1.77–17.28)	2.50 (1.97–3.16)	3.25 (3.04–3.47)	3.14 (2.95–3.35)
85 or older	6.85 (5.80–8.08)	3.50 (2.83–4.32)	4.49 (2.54–7.94)	7.98 (2.51–25.34)	3.43 (2.70–4.36)	4.63 (4.33–4.95)	4.52 (4.23–4.82)
Gender							
Women vs Men	1.00 (0.91–1.10)	0.89 (0.79–1.01)	0.50 (0.36–0.68)	0.73 (0.43–1.26)	0.95 (0.82–1.10)	0.91 (0.88–0.95)	0.91 (0.88–0.95)
Race/ethnicity							
NH-Whites	1.0 (ref)	1.0 (ref)	1.0 (ref)	1.0 (ref)	1.0 (ref)	1.0 (ref)	1.0 (ref)
NH-Blacks	1.30 (1.13–1.50)	1.75 (1.48–2.08)	0.87 (0.47–1.60)	0.65 (0.23–1.88)	1.09 (0.85–1.38)	1.24 (1.16–1.32)	1.25 (1.17–1.32)
NH-Asians/Pacific Islanders	1.00 (0.83–1.21)	0.83 (0.63–1.10)	0.70 (0.37–1.33)	0.29 (0.07–1.25)	0.46 (0.33–0.66)	0.93 (0.86–1.01)	0.92 (0.85–0.99)
Hispanics	1.30 (1.11–1.53)	1.10 (0.87–1.38)	0.92 (0.53–1.60)	0.62 (0.22–1.79)	0.53 (0.39–0.73)	1.03 (0.96–1.11)	1.03 (0.96–1.10)
Others	0.38 (0.09–1.51)	0.97 (0.31–3.03)	0 (0–0)	3.30 (0.43–25.07)	0.88 (0.28–2.77)	0.99 (0.72–1.37)	0.95 (0.69–1.30)
Unknown/missing	0.52 (0.22–1.26)	0.66 (0.21–2.08)	0 (0–0)	1.67 (0.20–13.68)	0.60 (0.19–1.90)	0.59 (0.41–0.85)	0.58 (0.40–0.82)
Marital status							
Married	1.0 (ref)	1.0 (ref)	1.0 (ref)	1.0 (ref)	1.0 (ref)	1.0 (ref)	1.0 (ref)
Unmarried	1.11 (1.01–1.23)	1.33 (1.17–1.52)	1.05 (0.76–1.46)	0.90 (0.51–1.59)	1.05 (0.91–1.22)	1.30 (1.24–1.35)	1.29 (1.24–1.34)
Unknown	1.00 (0.83–1.20)	0.96 (0.73–1.25)	0.94 (0.50–1.78)	0.91 (0.33–2.52)	1.08 (0.81–1.44)	1.12 (1.03–1.21)	1.11 (1.03–1.20)
Tumor stage							
Local	1.0 (ref)	1.0 (ref)	1.0 (ref)	1.0 (ref)	1.0 (ref)	1.0 (ref)	1.0 (ref)
Regional	0.98 (0.89–1.08)	1.11 (0.97–1.26)	1.02 (0.73–1.44)	1.16 (0.64–2.11)	0.99 (0.85–1.16)	1.11 (1.06–1.16)	1.11 (1.06–1.15)
Distant	0.82 (0.64–1.05)	0.97 (0.76–1.25)	0.38 (0.12–1.24)	0.29 (0.04–2.23)	0.81 (0.61–1.08)	1.38 (1.30–1.47)	1.34 (1.26–1.42)
Unknown/Missing	1.56 (1.26–1.94)	1.40 (1.06–1.85)	1.03 (0.42–2.57)	4.53 (1.88–10.92)	1.20 (0.84–1.72)	1.65 (1.52–1.79)	1.62 (1.49–1.75)
Tumor grade							
Well-differentiated	1.0 (ref)	1.0 (ref)	1.0 (ref)	1.0 (ref)	1.0 (ref)	1.0 (ref)	1.0 (ref)
Moderately-differentiated	1.04 (0.90–1.20)	1.18 (0.96–1.46)	1.37 (0.80–2.36)	0.73 (0.34–1.60)	0.98 (0.77–1.24)	1.06 (0.99–1.13)	1.05 (0.98–1.12)
Poorly-differentiated	1.01 (0.85–1.20)	1.24 (0.97–1.59)	0.91 (0.45–1.82)	0.66 (0.24–1.80)	1.24 (0.94–1.62)	1.10 (1.01–1.18)	1.10 (1.02–1.19)
Unknown/Missing	1.11 (0.93–1.32)	1.38 (1.08–1.77)	1.63 (0.87–3.05)	0.69 (0.26–1.85)	0.98 (0.73–1.30)	1.11 (1.03–1.20)	1.12 (1.03–1.21)
Tumor site							
Rectal vs. Colon	0.93 (0.83–1.05)	0.85 (0.72–0.99)	1.11 (0.76–1.61)	2.05 (1.15–3.64)	0.90 (0.74–1.08)	0.98 (0.94–1.03)	0.97 (0.92–1.02)
Chemotherapy							
Yes vs. No	0.73 (0.64–0.84)	0.58 (0.48–0.70)	0.70 (0.44–1.11)	1.06 (0.52–2.16)	0.92 (0.76–1.12)	0.80 (0.76–0.84)	0.80 (0.76–0.84)
Radiotherapy							
Yes vs. No	1.11 (0.91–1.36)	0.97 (0.73–1.30)	0.74 (0.36–1.52)	0.60 (0.21–1.69)	0.88 (0.64–1.22)	0.98 (0.91–1.07)	1.00 (0.93–1.09)
Comorbidity Scores							
0	1.0 (ref)	1.0 (ref)	1.0 (ref)	1.0 (ref)	1.0 (ref)	1.0 (ref)	1.0 (ref)
1	1.21 (1.10–1.32)	1.31 (1.15–1.49)	1.05 (0.76–1.43)	1.42 (0.80–2.53)	1.05 (0.90–1.22)	1.33 (1.27–1.38)	1.31 (1.26–1.36)
≥2	1.49 (1.33–1.67)	1.65 (1.42–1.92)	0.89 (0.57–1.41)	2.08 (1.07–4.04)	1.27 (1.06–1.52)	1.95 (1.86–2.05)	1.92 (1.84–2.01)

^a^ Hazard ratios adjusted for the following variables: age, gender, race/ethnicity, marital status, tumor stage, tumor grade, tumor site, comorbidity score, chemotherapy, radiation therapy, year of diagnosis, and SEER areas.

^b^ ADRD, Alzheimer's disease and related dementia; AD, Alzheimer's disease; Vascular, vascular dementia; DLB, dementia with Lewy bodies; FTD, Frontotemporal degeneration and dementia; MCI, Mild cognitive impairment, others (other dementia), and total (any of above ADRD).

### Risk of ADRD by considering death as a competing risk

Table 5 presents the hazard ratio of ADRD by antihypertensive medication adherence groups while considering death as a competing risk in regression models. As compared to those receiving angiotensin-II stimulating drugs with high adherence, the risk of AD, vascular dementia, and other types of ADRD were significantly higher in patients who received angiotensin-II inhibiting drugs regardless of high (1.12, 1.07–1.17 for AD) or low adherence (1.09, 1.03–1.16 for AD). The risk of AD and other types of ADRD was significantly lower in patients receiving a combination of angiotensin-II stimulating and inhibiting drugs, and significantly higher in those receiving other categories of antihypertensive drugs regardless of high or low adherence and in those with hypertension without receiving antihypertensive medications. Another interesting finding in this Find and Gray regression model considering death as a competing risk was that patients without hypertension now had a significantly higher risk of AD and other types of ADRD than those with hypertension who received angiotensin-II stimulating drugs with higher adherence. The risk of AD and other types of ADRD increased significantly with age and was higher in NH-black patients, those unmarried subjects, those with higher tumor stage or grade or rectal site, and those with higher comorbidity scores than their counterparts. The risk of AD and other types of ADRD was lower in women and in those receiving chemotherapy, but did not significantly vary by the receipt of radiation therapy.

**Table 5:** Adjusted hazard ratio of developing dementia (ADRD^a^) by drug group^a^ adherence and by considering death as competing risk.

	Hazard ratio (95% confidence intervals)^a^ of ADRD^b^						
Characteristics	AD^a^	Vascular	DLB^a^	FTD^a^	MCI^a^	Others	Total
Antihypertensive drug types							
Angiotensin-II stimulating drugs							
With high adherence	1.0 (ref)	1.0 (ref)	1.0 (ref)	1.0 (ref)	1.0 (ref)	1.0 (ref)	1.0 (ref)
With low adherence	0.94 (0.87–1.02)	0.95 (0.88–1.03)	0.93 (0.86–1.01)	0.94 (0.87–1.01)	0.93 (0.86–1)	0.97 (0.90–1.05)	0.97 (0.91–1.05)
Angiotensin-II inhibiting drugs							
With high adherence	1.12 (1.07–1.17)	1.13 (1.09–1.18)	1.13 (1.08–1.18)	1.12 (1.08–1.17)	1.12 (1.07–1.17)	1.16 (1.11–1.20)	1.15 (1.11–1.20)
With low adherence	1.09 (1.03–1.16)	1.08 (1.02–1.15)	1.08 (1.01–1.15)	1.08 (1.01–1.15)	1.07 (1.01–1.14)	1.10 (1.04–1.17)	1.10 (1.04–1.17)
Angiotensin -II stimulating + inhibiting drugs							
With high adherence	0.89 (0.85–0.93)	0.89 (0.85–0.93)	0.87 (0.83–0.91)	0.87 (0.83–0.91)	0.87 (0.83–0.91)	0.94 (0.90–0.98)	0.94 (0.90–0.98)
With low adherence	0.73 (0.67–0.79)	0.72 (0.66–0.78)	0.69 (0.64–0.76)	0.69 (0.64–0.76)	0.70 (0.64–0.76)	0.82 (0.76–0.89)	0.82 (0.76–0.88)
Other antihypertensives							
With high adherence	1.38 (1.30–1.47)	1.40 (1.32–1.48)	1.38 (1.30–1.46)	1.38 (1.30–1.46)	1.38 (1.30–1.46)	1.40 (1.32–1.48)	1.40 (1.32–1.48)
With low adherence	1.30 (1.22–1.38)	1.30 (1.22–1.39)	1.30 (1.22–1.39)	1.30 (1.22–1.39)	1.30 (1.22–1.39)	1.27 (1.20–1.35)	1.27 (1.19–1.35)
Did not receive antihypertensives	1.56 (1.50–1.63)	1.57 (1.50–1.63)	1.57 (1.51–1.64)	1.57 (1.50–1.63)	1.55 (1.49–1.62)	1.50 (1.44–1.56)	1.49 (1.43–1.55)
Did not have hypertension	1.34 (1.27–1.40)	1.33 (1.27–1.40)	1.34 (1.28–1.41)	1.34 (1.28–1.41)	1.33 (1.27–1.39)	1.25 (1.20–1.31)	1.24 (1.18–1.29)
Age (years)							
65–69	1.0 (ref)	1.0 (ref)	1.0 (ref)	1.0 (ref)	1.0 (ref)	1.0 (ref)	1.0 (ref)
70–74	1.27 (1.22–1.32)	1.25 (1.21–1.30)	1.25 (1.21–1.30)	1.25 (1.20–1.30)	1.25 (1.21–1.30)	1.30 (1.25–1.35)	1.30 (1.25–1.35)
75–79	1.60 (1.55–1.67)	1.57 (1.51–1.63)	1.56 (1.50–1.62)	1.56 (1.50–1.62)	1.57 (1.51–1.63)	1.66 (1.61–1.73)	1.67 (1.61–1.73)
80–84	2.13 (2.05–2.21)	2.07 (1.99–2.15)	2.05 (1.98–2.13)	2.06 (1.98–2.14)	2.07 (1.99–2.15)	2.23 (2.15–2.31)	2.23 (2.15–2.31)
85 or older	2.92 (2.82–3.04)	2.85 (2.74–2.95)	2.84 (2.73–2.95)	2.84 (2.74–2.95)	2.85 (2.75–2.96)	3.06 (2.95–3.17)	3.06 (2.96–3.18)
Gender							
Women vs. Men	0.81 (0.79–0.83)	0.80 (0.79–0.82)	0.80 (0.78–0.82)	0.80 (0.78–0.82)	0.81 (0.79–0.83)	0.84 (0.82–0.86)	0.84 (0.83–0.86)
Race/ethnicity							
NH-Whites	1.0 (ref)	1.0 (ref)	1.0 (ref)	1.0 (ref)	1.0 (ref)	1.0 (ref)	1.0 (ref)
NH-Blacks	1.15 (1.10–1.19)	1.15 (1.11–1.19)	1.14 (1.09–1.18)	1.13 (1.09–1.18)	1.13 (1.09–1.18)	1.15 (1.11–1.19)	1.15 (1.11–1.20)
NH-Asians/Pacific Islanders	0.75 (0.72–0.79)	0.74 (0.70–0.78)	0.73 (0.70–0.77)	0.74 (0.70–0.77)	0.73 (0.70–0.77)	0.79 (0.75–0.83)	0.79 (0.75–0.83)
Hispanics	0.97 (0.93–1.02)	0.96 (0.92–1.01)	0.96 (0.92–1.00)	0.96 (0.92–1.00)	0.95 (0.91–0.99)	0.99 (0.95–1.03)	0.99 (0.95–1.03)
Others	1.03 (0.87–1.23)	1.06 (0.90–1.26)	1.06 (0.89–1.26)	1.07 (0.90–1.27)	1.07 (0.90–1.26)	1.01 (0.86–1.19)	1.00 (0.85–1.19)
Unknown/missing	0.18 (0.12–0.26)	0.17 (0.12–0.25)	0.16 (0.10–0.23)	0.16 (0.11–0.24)	0.17 (0.12–0.25)	0.29 (0.22–0.38)	0.30 (0.23–0.39)
Marital status							
Married	1.0 (ref)	1.0 (ref)	1.0 (ref)	1.0 (ref)	1.0 (ref)	1.0 (ref)	1.0 (ref)
Unmarried	1.19 (1.16–1.22)	1.20 (1.17–1.23)	1.19 (1.16–1.22)	1.20 (1.17–1.23)	1.19 (1.16–1.22)	1.21 (1.19–1.24)	1.21 (1.19–1.24)
Unknown	1.03 (0.98–1.08)	1.03 (0.98–1.09)	1.03 (0.98–1.08)	1.03 (0.98–1.08)	1.03 (0.97–1.08)	1.05 (1.00–1.11)	1.05 (1.00–1.10)
Tumor stage							
Local	1.0 (ref)	1.0 (ref)	1.0 (ref)	1.0 (ref)	1.0 (ref)	1.0 (ref)	1.0 (ref)
Regional	1.71 (1.66–1.76)	1.75 (1.70–1.81)	1.77 (1.72–1.83)	1.78 (1.72–1.83)	1.75 (1.70–1.80)	1.57 (1.53–1.61)	1.56 (1.51–1.60)
Distant	6.94 (6.73–7.17)	7.10 (6.87–7.33)	7.31 (7.08–7.55)	7.34 (7.10–7.58)	7.07 (6.85–7.30)	5.20 (5.04–5.36)	5.05 (4.90–5.20)
Unknown/Missing	3.56 (3.40–3.72)	3.63 (3.46–3.80)	3.71 (3.54–3.89)	3.74 (3.57–3.92)	3.62 (3.45–3.79)	3.05 (2.92–3.19)	2.98 (2.85–3.12)
Tumor grade							
Well-differentiated	1.0 (ref)	1.0 (ref)	1.0 (ref)	1.0 (ref)	1.0 (ref)	1.0 (ref)	1.0 (ref)
Moderately-differentiated	1.12 (1.07–1.18)	1.14 (1.08–1.19)	1.13 (1.08–1.19)	1.13 (1.08–1.19)	1.12 (1.07–1.18)	1.11 (1.06–1.16)	1.11 (1.06–1.16)
Poorly-differentiated	1.46 (1.39–1.54)	1.49 (1.41–1.56)	1.48 (1.41–1.56)	1.49 (1.41–1.57)	1.47 (1.40–1.55)	1.39 (1.33–1.46)	1.39 (1.33–1.46)
Unknown/Missing	1.62 (1.54–1.71)	1.64 (1.55–1.72)	1.64 (1.56–1.73)	1.64 (1.56–1.73)	1.62 (1.54–1.71)	1.49 (1.42–1.56)	1.48 (1.41–1.56)
Tumor site							
Rectal vs. Colon	1.12 (1.09–1.15)	1.12 (1.09–1.16)	1.13 (1.10–1.16)	1.13 (1.10–1.17)	1.13 (1.09–1.16)	1.09 (1.06–1.12)	1.08 (1.05–1.11)
Chemotherapy							
Yes vs. No	0.62 (0.60–0.64)	0.62 (0.60–0.63)	0.62 (0.60–0.64)	0.62 (0.60–0.64)	0.62 (0.61–0.64)	0.65 (0.63–0.67)	0.65 (0.64–0.67)
Radiotherapy							
Yes vs. No	0.97 (0.93–1.02)	0.97 (0.92–1.01)	0.97 (0.93–1.01)	0.97 (0.92–1.01)	0.97 (0.92–1.01)	0.97 (0.93–1.01)	0.97 (0.93–1.01)
Comorbidity Scores							
0	1.0 (ref)	1.0 (ref)	1.0 (ref)	1.0 (ref)	1.0 (ref)	1.0 (ref)	1.0 (ref)
1	1.17 (1.14–1.19)	1.16 (1.13–1.19)	1.16 (1.13–1.19)	1.16 (1.13–1.19)	1.16 (1.13–1.19)	1.19 (1.16–1.22)	1.19 (1.16–1.22)
≥2	1.58 (1.53–1.62)	1.58 (1.54–1.63)	1.58 (1.54–1.63)	1.58 (1.54–1.63)	1.58 (1.53–1.62)	1.63 (1.59–1.68)	1.63 (1.59–1.68)

^a^ Hazard ratios adjusted for the following variables: age, gender, race/ethnicity, marital status, tumor stage, tumor grade, tumor site, comorbidity score, chemotherapy, radiation therapy, year of diagnosis, and SEER areas.

^b^ ADRD, Alzheimer's disease and related dementia; AD, Alzheimer's disease; Vascular, vascular dementia; DLB, dementia with Lewy bodies; FTD, Frontotemporal degeneration and dementia; MCI, Mild cognitive impairment; others (other dementia), and total (any of above ADRD).

### Sensitivity analyses assessing potential reverse causation bias and immortal time bias

In order to assess the impact of potential reverse causation bias and immortal time bias on the results, we performed sensitivity analyses by excluding incident ADRD cases that occurred during the first 3 and 5 years after the date of cancer diagnosis (Supplementary Table S4). The magnitude and direction of the adjusted hazard ratios of developing AD or ADRD were similar to those in Table 3 that did not exclude those cases within 3 or 5 years. For example, after excluding any ADRD cases that occurred during the first 5 years, those who received angiotensin-II inhibiting antihypertensives were significantly more likely to develop AD (1.26, 1.04–1.52), vascular dementia (1.35, 1.01–1.81), and total ADRD (1.22, 1.02–1.45) than patients who received angiotensin-II stimulating antihypertensive drugs. In addition, we performed a sensitivity analysis assessing the effect of renin-angiotensin system (RAS)-acting agents separately on the risk of AD. The hazard ratio of AD for patients receiving angiotensin-converting enzyme inhibitors (0.98, 0.83–1.14) and for those receiving direct renin inhibitors (0.94, 0.64–1.40) was not significantly different as compared to those receiving angiotensin-receptor blockers.

## Discussion

This study examined the effects of several classes of antihypertensive medications on the risk of AD and ADRD in a large cohort of men and women with colorectal cancer. The study found that patients with hypertension who received angiotensin-II inhibiting antihypertensives were significantly more likely to develop AD, vascular dementia and total ADRD than those who received angiotensin-II stimulating antihypertensive drugs. Patients who received a combination of angiotensin-II stimulating and inhibiting antihypertensive drugs did not have significantly different risks of ADRD, but those who received other types of antihypertensive drugs or did not receive antihypertensive drugs for hypertension had significantly higher risks of AD and ADRD. These findings remained unchanged overall after considering death as a competing risk or after adjusting for other confounders. Patients with colorectal cancer who did not have hypertension had a significantly lower risk of AD and ADRD.

Our findings on the contrasting risk of AD and ADRD between angiotensin-II stimulating vs. inhibiting antihypertensive drugs in a large cohort of men and women with colorectal cancer support the results and conclusions of previous studies by van Dalen et al. (10), Marcum et al. (11), and other researchers (12–22). Although each study population was different, the magnitude and direction of the risk of AD and ADRD associated with angiotensin-II stimulating or inhibiting antihypertensive medications were consistent.

Additional unique findings from our study were that patients with hypertension had a significantly higher risk of AD and ADRD when having a low adherence to angiotensin-II stimulating antihypertensive medications or with high or low adherence to angiotensin-II inhibiting drugs vs. those with a high adherence to angiotensin-II stimulating medications. Some previously proposed mechanisms regarding how the risk of AD and ADRD is lowered in association with angiotensin-II stimulating antihypertensive drug use include beneficial effects of reduced ischemia, enhancement of cerebral blood flow, and improvement of spatial memory processing (10, 11, 28–31).

Our study also demonstrated that patients with hypertension who did not receive antihypertensive medications had a higher risk of ADRD and those who did not have hypertension actually had a lower risk of both AD and ADRD. These findings are in line with previous studies (6–9, 40, 41), which demonstrated that hypertension is a major risk factor for ADRD. As a modifiable risk for ADRD, having hypertension treated and well-controlled blood pressure is expected to reduce the risk of ADRD. A meta-analysis of 14 randomized clinical trials concluded that lowering blood pressure with antihypertensive agents is significantly associated with a lower risk of incident dementia or cognitive impairment when compared to the control group (42). Another meta-analysis that summarized 6 prospective community-based studies in dementia-free adults aged ≥55 found that in the high blood pressure stratum, those receiving any antihypertensive medications had a reduced risk for developing AD (hazard ratio: 0.84, 95% CI: 0.73–0,97, *p* = 0.021) and for developing other dementia (0.88, 0.79–0.98, *p* = 0.019) as compared with those who did not receive any antihypertensive medications (43). There was no significant association, however, between the receipt of antihypertensive medications and the risk of AD or other dementia among those with normal blood pressure (6–9).

Our study also showed that patients without hypertension and those with hypertension who did not receive antihypertensive medications or who received medications other than angiotensin-II stimulating drugs had a significantly higher risk of AD and other types of ADRD as compared to those with hypertension who received angiotensin-II stimulating drugs with higher adherence after considering death as a competing risk in the Find and Gray regression models. This finding might indicate that using angiotensin-II stimulating medications to treat hypertension could help reduce the risk of AD and ADRD regardless of age, which by itself is associated with a significantly increased risk of ADRD and mortality. Importantly, the results of this study also revealed another potential mechanism by which ADRD risk is reduced. Specifically, we found that cancer chemotherapy is associated with a reduced risk for AD and ADRD. This lends support to similar past findings (5, 44).

There are several limitations to be noted in this study. First, the classification of antihypertensive medications and adherence were based on the 12 months after the drug initiation. The changes or switches to different medications and related medication adherence status over the long-term follow-up periods were not factored into the analysis. Second, the study outcomes on ADRD were defined from diagnostic codes in Medicare claims data that may be subject to potential overestimation or underestimation, even though Medicare data were reported to have a sensitivity of 85% and a specificity of 89% for identifying overall ADRD (45, 46). Third, in order to obtain complete information on antihypertensive medications, comorbidities and outcomes, the study only included patients who had Medicare Part-A (inpatient), Part-B (outpatient and physician office visits), Part-D (comprehensive drug coverage), and no Part-C (Medicare Advantage) or Health Maintenance Organization in Medicare beneficiaries aged ≥65. The study findings may not be generalizable to other populations with different Medicare plans or patients of <65 years. Fourth, the study datasets did not consist of all relevant variables, including smoking, education, income, social network, and family support, which could have affected the magnitude or directions of the estimated associations in this study. Fifth, we did a sensitivity analysis on renin-angiotensin system (RAS)-acting agents and found that the risk of AD in patients receiving angiotensin-converting enzyme inhibitors or direct renin inhibitors was not significantly different from those receiving angiotensin-receptor blockers. However, because of the scope and focus of this manuscript on the risk of AD and other types of ADRD between patients receiving angiotensin II–stimulating antihypertensive medications and those receiving angiotensin II–inhibiting antihypertensive medications, we were unable to address antihypertensive drugs acting in the central or peripheral nervous system and their effects on the risk of ADRD (47), which need to be explored further in future studies. Sixth, because Alzheimer's disease and other dementia are neurodegenerative conditions that take a long time to develop. Study period for subjects in 2007–2015 with follow-up to 2016 may be relatively short. Longer follow-up period would be helpful to examine the potentially increasing gaps in the long-term risk of Alzheimer's disease and other dementia by different classes of antihypertensive drugs.

In conclusion, the risk of AD and ADRD in patients with hypertension who received angiotensin-II inhibiting antihypertensive medications was higher than in those receiving angiotensin-II stimulating antihypertensive drugs in men and women with colorectal cancer. Adherence to taking antihypertensive medications appeared to affect the risk of ADRD. The risk of ADRD was not significantly associated with receiving a combination of angiotensin-II stimulating and inhibiting antihypertensive drugs and was higher in those with hypertension who received other types of antihypertensive drugs or did not receive antihypertensive medication. Further studies would be helpful to examine the effects of specific antihypertensive drug changes on the findings during longitudinal follow-up and to confirm our findings in other populations.

## Data Availability

The datasets presented in this article are not readily available because The National Cancer Institute's SEER (Surveillance, Epidemiology, and End Results)-Medicare Data User Agreement (DUA) specifically requests that “You (the Investigators) will not permit others to use the data except for collaborators within your institution involved with the research as described in your proposal”. Requests to access the datasets should be directed to SEERMedicare@imsweb.com.
