# Supplementary material for: Angiotensin-II stimulating vs. inhibiting antihypertensive drugs and the risk of Alzheimer's disease or related dementia in a large cohort of older patients with colorectal cancer
Source: Front Cardiovasc Med. 2023 May 5;10:1136475. doi: 10.3389/fcvm.2023.1136475 (PMC10196474; doi:10.3389/fcvm.2023.1136475)

Supplemental Materials:

Table S1. Antihypertensive Medication Classes and Common Generic and Brand Names

#	Antihypertensive medication classes FDA Chronic Antihypertensive Treatment Classes	Generic Name (GNN)	Brand Name (BN)*
**Category-1: Angiotensin II Stimulating drugs**			
1	Angiotensin II receptor blockers (**ARB**)	Azilsartan<br>Candesartan<br>Eprosartan<br>Irbesartan<br>Losartan<br>Olmesartan<br>Telmisartan<br>Valsartan	Edarbi, Edarbychlor‡<br>Atacand, Atacand HCT‡<br>Teveten, Teveten HCT‡, Teveten Plus‡<br>Avapro, Avalide‡<br>Cozaar, Hyzaar‡<br>Benicar, Azor‡, Benicar HCT‡, Tribenzor‡<br>Micardis, Micardis HCT‡, Micardis Plus‡,Twynsta‡<br>Diovan, Prexxartan§, Byvalson‡, Diovan HCT‡, Exforge HCT‡, Entresto‡, Valturna‡
2	Calcium channel blocker (CCB)- dihydropyridine	Amlodipine<br>Felodipine<br><br><br>Isradipine<br>Nicardipine<br>Nifedipine<br><br>Nimodipine<br>Nisoldipine	Norvasc, Amturnide‡, Azor‡, Exforge HCT‡, Lotrel‡, Prestalia‡, Tekamlo‡, Tribenzor‡, Twynsta‡<br>Cabren, Cardioplen XL, Felendil XL, Felogen XL, Felotens XL, Keloc SR, Neofel XL, Plendil, Renedil, Vascalpha, Lexxel‡§<br><br>Cardene, Cardene SR<br>Adalat, Adalat CC, Afeditab CR, Nifediac CC, Nifedical XL, Procardia, Procardia XL,<br>Sular
3	Diuretics: thiazide	Bendroflumenthiazide<br>Chlorothiazide<br>Hydrochlorothiazide<br><br>Hydroflumethiazide<br>Polythiazide<br>Trichlormethiazide	Naturetin§, NeoNaClex§, Urizide§, Corzide‡<br>Diuril, Tekturna HCT‡<br>Microzide, Accuretic‡, Aldactazide‡, Atacand HCT‡, Avalide‡, Benicar HCT‡, Capozide‡, Corzide‡, Dutoprol‡, Exforge HCT‡, Hydra-zide‡, Hyzaar‡, Inderide‡, Lopressor HCT‡, Lotensin HCT‡, Maxide‡, Micardis HCT‡, Micardis Plus‡, Moduretic‡, Teveten HCT‡, Teveten Plus‡, Tribenzor‡, Uniretic‡, Vaseretic‡, Zestoretic‡, Ziac‡, Diucardin; Renese; Achletin, Diu-Hydrin and Triflumen
4	Diuretics: thiazide-type	Chlorthalidone<br>Indapamide<br>Metolazone	Hygroton, Thalitone, Chlorthalid, Clorpres‡, Edarbychlor‡, Tenoretic‡<br>Zaroxolyn
**Category-2: Angiotensin II Inhibiting drugs**			
5	Angiotensin-converting enzyme (**ACE**) inhibitor	Benazepril<br>Captopril<br>Enalapril<br>Fosinopril<br>Lisinopril<br>Moexipril<br>Perindopril<br>Quinapril<br>Ramipril<br>Trandolapril	Lotensin, Lotensin HCT‡, Lotrel‡<br>Capoten, Captoril, Capozide‡<br>Vasotec, Enalaprilat, Epaned, Lexxel‡§, Vaseretic‡<br>Monopril<br>Prinivil, Qbrelis, Zestril, Zestoretic‡<br>Univasc, Uniretic‡<br>Aceon, Prestalia‡<br>Accupril, Accuretic‡<br>Altace<br>Mavik, Tarka‡
6	Beta adrenergic blockers (**β-blockers**)	Acebutolol<br>Atenolol<br>Betaxolol<br>Bisoprolol	Sectral<br>Tenormin, Tenoretic‡<br>Kerlone<br>Monocor, Zebeta, Ziac‡<br>Coreg, Coreg CR

		Carvedilol {*also with α1 properties*}<br>Esmolol⊓<br>Labetalol {*also with α1 properties*}<br>Metoprolol<br><br>Nadolol<br>Nebivolol<br>Penbutolol<br>Propranolol<br>Sotalol<br><br>Carteolol<br>Pindolol<br>Timolol	Brevibloc<br>Trandate<br>Kapspargo Sprinkle, Lopressor, Toprol XL, Dutoprol‡, Lopressor HCT‡<br>Corgard, Corzide‡<br>Bystolic, Byvalson‡<br>Levatol<br>Inderal, Inderal LA, InnoPran XL, Inderide‡<br>Betapace, Sorine⊓<br><br><br>Ocupress;<br>Visken;<br>Blocadren, Timoptic-xe, Istalol, Timoptic, Betimol, and Timoptic Ocudose (PF)
7	Calcium channel blocker – non-dihydropyridine	Diltiazem<br><br><br>Verapamil	Cardizem, Cardizem CD, Cardizem LA, Dilacor, Dilacor XR, Dilatrate, Diltazem, Diltazem CD, Diltiaz, Diltiaz CD, Diltiaz SR, Tiazac<br>Calan, Calan HS, Calan SR, Covera HS, Isoptin, Isoptin SR, Verelan, Verelan PM, Tarka‡
**Category-3: Other types of anti-hypertensive drugs**			
8	Aldosterone antagonists †	Eplerenone<br>Spironolactone	Inspra<br>Aldactone, Carospir, Aldactazide‡
9	Alpha 1 adrenergic receptor agonist (selective; **α-blocker**)	Doxazosin<br>Prazosin<br>Terazosin	Cardura (XL)<br>Minipress, Prazin, Prazo<br>Hytrin
10	Arteriolar vasodilators	Hydralazine<br>Minoxidil	-, Hydra-zide‡<br>Loniten, Minodyl, Minoxidil HTN
11	Autonomic ganglionic vasodilators	Mecamylamine	-
12	Central alpha 2 adrenergic agonists	Clonidine<br>Guanabenz§<br>Guanfacine<br>Methyldopa	Catapres, Jenloga, Kapvay, Nexiclon XR, Clorpres‡<br>Wytensin§<br>Intuniv, Tenex<br>Aldomet
13	Diuretics: loop	Bumetanide<br>Ethacrynic acid<br>Furosemide<br>Torsemide	Bumex, Burinex<br>Edecrin⊓<br>Lasix<br>Demadex
14	Diuretics: potassium-sparing†	Amiloride<br>Triamterene	Midamor, Moduretic‡<br>Dyrenium, Maxide‡
15	Peripheral adrenergic neuron antagonist†	Reserpine	Serpasil
16	Renin inhibitors	Aliskiren	Tekturna, Tekturna HCT‡, Teklamo‡, Amturnide‡, Valturna‡

*Part-D data appear to have generic name (GNN) only because BN has the same info as GNN in the dataset (so far as noted).
†Not used alone/used in combination for chronic hypertension treatment
‡Combination medication also listed in another class
§Currently discontinued in the US

Source in part from:
Healthline: A List of Blood Pressure Medications. https://www.healthline.com/health/high-blood-pressure-hypertension-medication#overview, accessed July 19, 2022.

Table S2. ICD-9 and ICD-10 codes for Alzheimer's Disease and related Dementia (ADRD)

ICD-9	Description for ICD-9 codes	ICD-10	Description for ICD-10 codes
290.0	Senile dementia, uncomplicated	F03.90	Unspecified dementia without behavioral disturbance
290.1	Presenile dementia, uncomplicated	F03.90	Unspecified dementia without behavioral disturbance
290.2	Senile dementia with delusional or depressive features	F03.90	Unspecified dementia without behavioral disturbance
290.3	Senile dementia with delirium (acute confusional state)	F03.90	Unspecified dementia without behavioral disturbance
290.40	Vascular dementia	F01.50	Vascular dementia
290.41	Vascular dementia, with delirium	F01.51	Vascular dementia with behavioral disturbance
290.42	Vascular dementia, with delusions		
290.43	Vascular dementia, with depressed mood		
290.8	Other specified senile psychotic conditions	F03.90	Unspecified dementia without behavioral disturbance
290.9	Unspecified senile psychotic condition	F03.90	Unspecified dementia without behavioral disturbance
291.2	Alcohol-induced persisting dementia	F10.27	Alcohol-induced dementia
292.82	Drug-induced persisting dementia	F19.97	Other-psychoactive substance use/unspecified with psychoactive substance induced persisting dementia
294.10	Dementia in conditions classified elsewhere without behavioral disturbance	F02.80	Dementia in other diseases classified elsewhere without behavioral disturbance
294.11	Dementia in conditions classified elsewhere with behavioral disturbance	F02.81	Dementia in other diseases classified elsewhere with behavioral disturbance
294.20	Dementia, unspecified, without behavioral disturbance	F03.90	Unspecified dementia without behavioral disturbance
294.21	Dementia, unspecified, with behavioral disturbance	F03.91	Unspecified dementia with behavioral disturbance
294.8	Other persistent mental disorders due to conditions classified elsewhere	F06.0	Psychotic disorder with hallucinations due to known physiological condition
294.9	Unspecified persistent mental disorders due to conditions classified elsewhere	F06.8	Other specified mental disorders due to known physiological condition
331.0	Alzheimer's disease	G30	Alzheimer's disease
		G30.0	Alzheimer's disease with early onset
		G30.1	Alzheimer's disease with late onset
		G30.8	Other Alzheimer's disease
		G30.9	Alzheimer's disease, unspecified
331.11	Frontotemporal dementia, Pick's disease	G31.01	Frontotemporal dementia, Pick's disease
331.19	Other frontotemporal dementia	G31.09	Other frontotemporal dementia
331.2	Senile degeneration of brain	G31.1	Senile degeneration of brain, not elsewhere classified
331.7	Cerebral degeneration in dis. classified elsewhere	G94	Other disorders of brain in dis. classified elsewhere
331.82	Dementia with Lewy bodies	G31.83	Dementia with Lewy bodies
331.83	Mild cognitive impairment, so stated	G31.84	Mild cognitive impairment, so stated
331.89	Other cerebral degeneration	G31.89	Other specified degenerative dis. of nervous system
331.9	Cerebral degeneration, unspecified	G31.9	Degenerative disease of nervous system, unspecified
797	Senility without mention of psychosis	R41.81	Age-related cognitive decline

Table S3. Incidence-density of dementia (ADRD*) by antihypertensive drug types

	Incidence-density of ADRD (per 1,000 person-years)						
**Characteristics**	**AD***	**Vascular**	**DLB***	**FTD***	**MCI***	**Others**	**Total ADRD**
**Antihypertensive drug types**							
Angiotensin-II stimulating drugs	12.5	6.1	0.9	0.4	4.2	66.2	71.8
Angiotensin-II inhibiting drugs	13.3	7.5	1.2	0.4	5.4	79.6	85.5
Angiotensin-II stimulating + inhibiting drugs	14.4	7.5	0.9	0.4	4.8	70.7	76.2
Other antihypertensive drugs	16.3	10.6	1.2	0.3	6.6	111.9	120.3
Did not receive any antihypertensive drugs	11.0	6.4	1.1	0.2	5.0	87.2	93.8
Did not have hypertension	6.7	2.3	0.8	0.2	3.1	39.3	42.3
**Age (years)**							
65-69	4.2	2.8	0.4	0.1	2.3	29.0	31.8
70-74	7.5	4.1	0.8	0.3	3.3	46.5	50.6
75-79	13.4	7.2	1.4	0.4	5.0	72.7	79.0
80-84	20.6	10.3	1.4	0.4	6.7	112.7	120.0
85 or older	28.4	14.1	1.6	0.6	10.1	189.9	204.1
**Gender**							
Men	10.3	5.8	1.3	0.4	4.4	66.0	71.3
Women	14.1	7.3	0.8	0.3	5.1	78.1	83.9
**Race/ethnicity**							
NH-Whites	12.5	6.4	1.1	0.4	5.3	73.3	78.9
NH-Blacks	14.9	11.1	0.7	0.2	4.9	90.4	97.7
NH-Asians/Pacific Islanders	9.9	4.6	0.9	0.1	2.5	59.6	63.7
Hispanics	12.6	5.9	1.0	0.3	2.9	65.0	70.3
Others	2.7	4.1	0	1.3	4.1	54.2	56.0
Unknown/missing	5.0	3.0	0	1.0	3.0	31.4	33.8
**Marital status**							
Married	10.0	4.8	1.0	0.3	4.1	54.1	58.7
Unmarried	15.1	8.7	1.0	0.3	5.4	93.7	100.5
Unknown	12.7	6.0	1.0	0.4	5.0	70.0	75.3
**Tumor stage**							
In-situ/local stage	13.6	6.6	1.2	0.3	4.8	64.4	69.7
Regional	11.9	6.4	1.0	0.3	4.8	70.5	76.1
Distant	5.1	5.6	0.2	0.1	4.1	107.6	112.9
Unknown/Missing	19.3	12.3	1.0	1.5	7.1	169.8	180.0
**Tumor grade**							
Well-differentiated	12.4	5.6	0.9	0.5	4.7	62.6	68.0
Moderately-differentiated	12.6	6.4	1.1	0.3	4.5	69.8	74.8
Poorly-differentiated	12.0	7.1	0.6	0.3	6.2	82.0	88.7
Unknown/Missing	12.4	7.9	1.2	0.4	4.5	82.8	89.5
**Tumor site**							
Colon	13.2	7.2	1.0	0.3	5.1	76.1	82.1
Rectal	10.1	4.9	1.0	0.5	3.8	62.6	67.1
**Chemotherapy**							
No	14.4	7.8	1.2	0.4	5.3	81.1	87.3
Yes	7.1	3.4	0.6	0.3	3.5	50.4	54.5
**Radiotherapy**							
No	12.8	6.9	1.0	0.3	4.9	74.3	79.9
Yes	9.1	4.0	0.6	0.3	3.2	56.5	61.5
**Comorbidity Scores**							
0	10.4	5.0	1.0	0.3	4.3	53.8	58.3
1	13.9	7.5	1.1	0.4	4.9	79.6	85.4
≥2	16.4	10.2	0.9	0.5	6.1	127.5	136.8
**SEER Areas**							
Connecticut	16.6	15.0	1.6	0.6	7.0	85.0	93.1
Detroit	18.0	11.9	0.9	0.3	5.3	90.4	96.6
Hawaii	7.9	6.3	1.6	0	1.6	54.8	55.6
Iowa	11.3	4.9	0.8	0.1	4.9	66.1	69.6
New Mexico	6.9	4.3	0.8	0.3	4.0	54.2	58.0
Seattle	8.0	5.6	1.1	1.0	3.9	59.1	63.1
Utah	9.1	3.9	0	0	3.6	60.8	64.0
Georgia	10.7	4.4	0.6	0.2	3.5	64.2	68.7
Kentucky	14.1	5.4	0.8	0.1	2.9	78.3	82.4
Louisiana	12.6	6.6	0.8	0.4	3.7	77.5	81.9
New Jersey	17.3	8.9	1.4	0.4	6.0	82.7	90.9
California	10.4	5.3	1.0	0.3	5.2	69.8	75.8
**Total**	**12.5**	**6.6**	**1.0**	**0.3**	**4.8**	**72.7**	**78.3**

* ADRD (Alzheimer's disease and related dementia), AD (Alzheimer's disease), Vascular (vascular dementia), DLB (dementia with Lewy bodies), FTD (Frontotemporal degeneration and dementia), MCI (Mild cognitive impairment), others (other dementia), and total (any of above ADRD).

Table S4. Adjusted hazard ratio of developing dementia by antihypertensive drug types by excluding dementia cases that occurred within 3 and 5 years from the baseline

Characteristics	Hazard ratio (95% CI)* of ADRD by excluding dementia cases that occurred within 3 years from baseline			Hazard ratio (95% CI)* of ADRD by excluding dementia cases that occurred within 5 years from baseline		
	AD**	Vascular	Total ADRD**	AD**	Vascular	Total ADRD**
**Antihypertensive drug types**						
Angiotensin-II stimulating drugs	1.0 (ref)	1.0 (ref)	1.0 (ref)	1.0 (ref)	1.0 (ref)	1.0 (ref)
Angiotensin-II inhibiting drugs	1.26 (1.04-1.52)	1.35 (1.01-1.81)	1.25 (1.12-1.41)	1.20 (0.92-1.57)	1.34 (0.87-2.05)	1.22 (1.02-1.45)
Angiotensin -II stimulating + inhibiting drugs	1.09 (0.91-1.31)	1.26 (0.95-1.66)	1.23 (1.10-1.37)	1.03 (0.8-1.33)	1.37 (0.93-2.03)	1.14 (0.97-1.34)
Other antihypertensive drugs	1.36 (1.05-1.78)	1.72 (1.17-2.53)	1.31 (1.11-1.55)	1.07 (0.7-1.63)	1.88 (1.06-3.33)	1.17 (0.89-1.55)
Did not receive any antihypertensive drugs	1.19 (0.95-1.49)	0.82 (0.56-1.21)	1.01 (0.87-1.16)	1.03 (0.74-1.45)	0.63 (0.34-1.18)	0.93 (0.74-1.16)
Did not have hypertension	1.05 (0.82-1.34)	0.50 (0.31-0.81)	0.87 (0.75-1.01)	1.15 (0.83-1.59)	0.47 (0.24-0.92)	0.87 (0.70-1.08)
**Age (years)**						
65-69	1.0 (ref)	1.0 (ref)	1.0 (ref)	1.0 (ref)	1.0 (ref)	1.0 (ref)
70-74	1.60 (1.28-2.01)	1.70 (1.22-2.36)	1.58 (1.41-1.78)	1.79 (1.31-2.46)	2.01 (1.28-3.17)	1.44 (1.22-1.70)
75-79	3.16 (2.56-3.89)	2.83 (2.07-3.87)	2.45 (2.19-2.75)	3.93 (2.93-5.27)	3.17 (2.05-4.92)	2.39 (2.03-2.81)
80-84	4.77 (3.86-5.89)	3.09 (2.23-4.28)	3.24 (2.88-3.65)	5.43 (4.02-7.33)	3.26 (2.05-5.20)	3.12 (2.63-3.71)
85 or older	6.28 (5.04-7.83)	4.03 (2.87-5.67)	4.68 (4.12-5.31)	6.62 (4.77-9.17)	4.38 (2.67-7.18)	4.43 (3.66-5.38)
**Gender**						
Men	1.0 (ref)	1.0 (ref)	1.0 (ref)	1.0 (ref)	1.0 (ref)	1.0 (ref)
Women	1.01 (0.89-1.15)	0.91 (0.75-1.11)	0.91 (0.84-0.98)	0.98 (0.82-1.18)	0.91 (0.69-1.21)	0.97 (0.86-1.09)
**Race/ethnicity**						
NH-Whites	1.0 (ref)	1.0 (ref)	1.0 (ref)	1.0 (ref)	1.0 (ref)	1.0 (ref)
NH-Blacks	1.26 (1.03-1.54)	1.38 (1.03-1.86)	1.09 (0.96-1.24)	1.12 (0.84-1.51)	1.19 (0.77-1.85)	1.18 (0.97-1.42)
NH-Asians/Pacific Islanders	0.74 (0.55-0.98)	0.61 (0.38-0.98)	0.77 (0.66-0.90)	0.59 (0.39-0.90)	0.51 (0.24-1.09)	0.73 (0.58-0.92)
Hispanics	1.26 (1.01-1.57)	1.06 (0.75-1.51)	0.99 (0.87-1.14)	0.91 (0.65-1.28)	1.27 (0.80-2.02)	1.01 (0.82-1.23)
Others	0.33 (0.05-2.37)	0.70 (0.10-5.00)	0.79 (0.41-1.53)	-[T]	-	0.37 (0.09-1.51)
Unknown/missing	0.77 (0.29-2.09)	1.39 (0.44-4.43)	0.96 (0.58-1.59)	0.60 (0.15-2.45)	3.40 (1.04-11.14)	0.78 (0.35-1.79)
**Marital status**						
Married	1.0 (ref)	1.0 (ref)	1.0 (ref)	1.0 (ref)	1.0 (ref)	1.0 (ref)
Unmarried	1.03 (0.90-1.17)	1.11 (0.91-1.36)	1.14 (1.05-1.23)	1.01 (0.84-1.21)	1.00 (0.76-1.33)	1.04 (0.92-1.17)
Unknown	0.89 (0.69-1.16)	0.95 (0.64-1.40)	1.02 (0.87-1.19)	0.95 (0.66-1.36)	0.65 (0.35-1.20)	1.00 (0.79-1.27)
**Tumor stage**						
Local	1.0 (ref)	1.0 (ref)	1.0 (ref)	1.0 (ref)	1.0 (ref)	1.0 (ref)
Regional	0.97 (0.85-1.11)	1.05 (0.85-1.29)	1.02 (0.94-1.11)	0.89 (0.73-1.08)	1.25 (0.93-1.67)	1.02 (0.90-1.15)
Distant	0.80 (0.54-1.18)	1.12 (0.65-1.91)	1.00 (0.82-1.23)	0.79 (0.42-1.45)	1.50 (0.68-3.28)	1.21 (0.89-1.66)
Unknown/Missing	0.98 (0.66-1.47)	1.30 (0.74-2.29)	1.38 (1.10-1.73)	1.31 (0.77-2.21)	1.78 (0.85-3.71)	1.08 (0.73-1.60)
**Tumor grade**						
Well-differentiated	1.0 (ref)	1.0 (ref)	1.0 (ref)	1.0 (ref)	1.0 (ref)	1.0 (ref)
Moderately-differentiated	0.96 (0.80-1.17)	1.09 (0.80-1.48)	1.05 (0.93-1.19)	0.84 (0.65-1.08)	1.23 (0.79-1.91)	0.99 (0.83-1.17)
Poorly-differentiated	0.99 (0.79-1.25)	1.12 (0.77-1.61)	1.10 (0.96-1.28)	0.84 (0.60-1.15)	1.04 (0.60-1.79)	1.01 (0.82-1.25)
Unknown/Missing	1.03 (0.81-1.30)	1.07 (0.74-1.56)	1.00 (0.86-1.16)	0.96 (0.70-1.32)	1.12 (0.65-1.93)	1.01 (0.82-1.26)
**Tumor site**						
Colon	1.0 (ref)	1.0 (ref)	1.0 (ref)	1.0 (ref)	1.0 (ref)	1.0 (ref)
Rectal	0.81 (0.69-0.96)	0.84 (0.65-1.09)	0.95 (0.86-1.05)	0.84 (0.66-1.06)	0.83 (0.57-1.20)	0.95 (0.82-1.10)

**Chemotherapy**						
No	1.0 (ref)	1.0 (ref)	1.0 (ref)	1.0 (ref)	1.0 (ref)	1.0 (ref)
Yes	0.76 (0.64-0.91)	0.73 (0.56-0.97)	0.94 (0.85-1.04)	0.83 (0.64-1.07)	0.68 (0.46-0.99)	0.90 (0.77-1.05)
**Radiotherapy**						
No	1.0 (ref)	1.0 (ref)	1.0 (ref)	1.0 (ref)	1.0 (ref)	1.0 (ref)
Yes	1.11 (0.83-1.49)	0.83 (0.51-1.35)	0.94 (0.80-1.11)	0.97 (0.63-1.49)	1.06 (0.56-1.99)	0.97 (0.76-1.24)
**Comorbidity Scores**						
0	1.0 (ref)	1.0 (ref)	1.0 (ref)	1.0 (ref)	1.0 (ref)	1.0 (ref)
1	1.20 (1.05-1.35)	1.27 (1.04-1.54)	1.27 (1.17-1.37)	1.26 (1.05-1.50)	1.05 (0.80-1.38)	1.24 (1.10-1.39)
≥2	1.45 (1.23-1.71)	1.22 (0.94-1.59)	1.65 (1.50-1.83)	1.44 (1.13-1.83)	1.14 (0.78-1.66)	1.80 (1.55-2.09)

* Hazard ratios adjusted for the following variables: age, gender, race/ethnicity, marital status, tumor stage, tumor grade, tumor site, comorbidity score, chemotherapy, radiation therapy, year of diagnosis, and SEER areas.

** AD (Alzheimer's disease), Vascular (vascular dementia), total ADRD (Alzheimer's disease and related dementia) included AD, vascular dementia, DLB (dementia with Lewy bodies), FTD (Frontotemporal degeneration and dementia), MCI (Mild cognitive impairment), and other dementia.

[T] no cases.

Figure S1. Forest Plot on the hazard ratio of developing AD in patients receiving angiotensin II–inhibiting vs stimulating antihypertensive medications

[AD (Alzheimer's disease), Vascular (vascular dementia), DLB (dementia with Lewy bodies), FTD (Frontotemporal degeneration and dementia), MCI (Mild cognitive impairment), others (other dementia), and total ADRD (Alzheimer's disease and related dementia)]

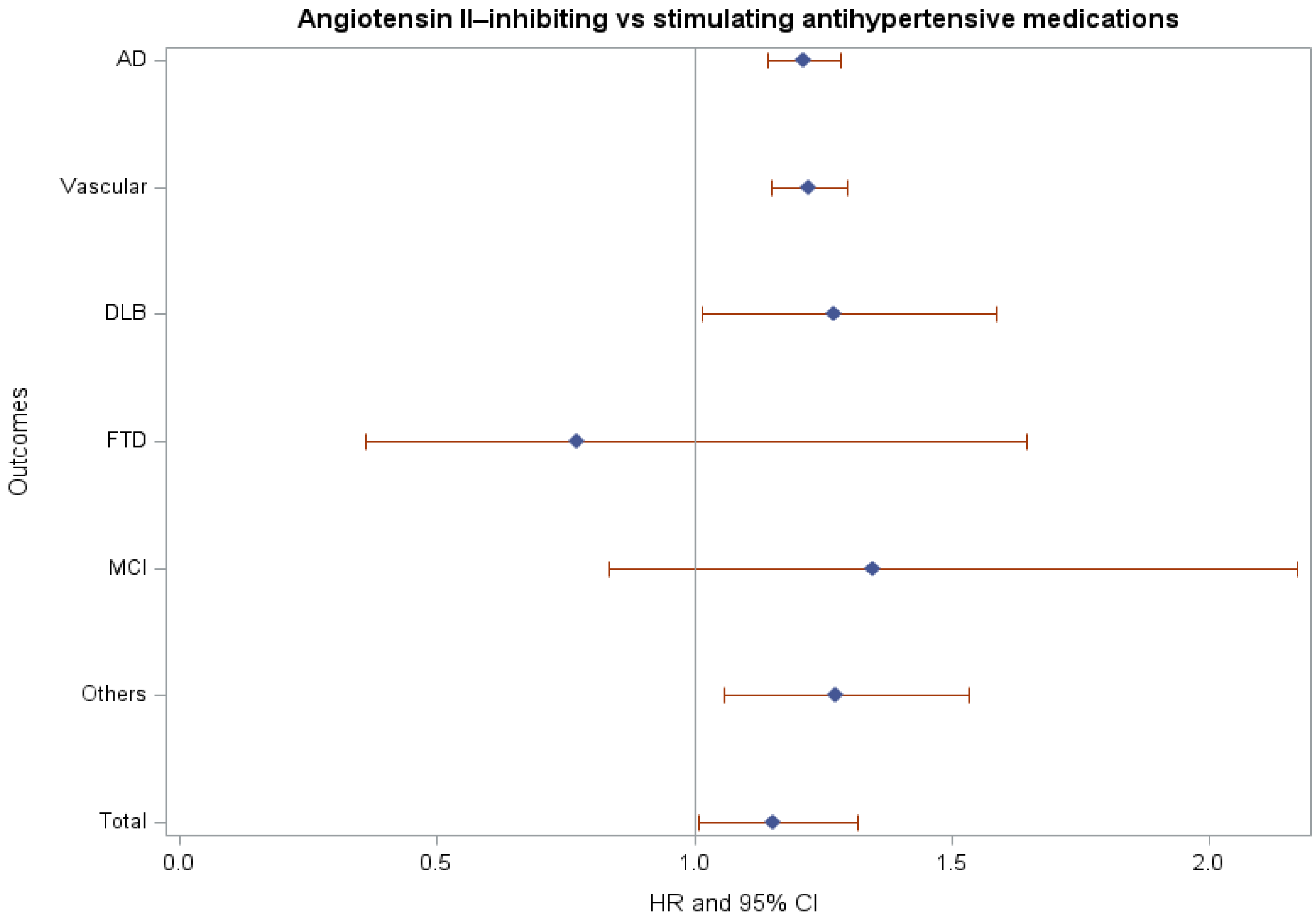

Supplement: Supplementary file 1 [file Datasheet1.pdf]
